# Gain of DNA methylation is enhanced in the absence of CTCF at the human *retinoblastoma *gene promoter

**DOI:** 10.1186/1471-2407-11-232

**Published:** 2011-06-10

**Authors:** Mercedes Dávalos-Salas, Mayra Furlan-Magaril, Edgar González-Buendía, Christian Valdes-Quezada, Erandi Ayala-Ortega, Félix Recillas-Targa

**Affiliations:** 1 Instituto de Fisiología Celular, Departamento de Genética Molecular, Universidad Nacional Autónoma de México, México D.F., México

## Abstract

**Background:**

Long-term gene silencing throughout cell division is generally achieved by DNA methylation and other epigenetic processes. Aberrant DNA methylation is now widely recognized to be associated with cancer and other human diseases. Here we addressed the contribution of the multifunctional nuclear factor CTCF to the epigenetic regulation of the human *retinoblastoma *(*Rb*) gene promoter in different tumoral cell lines.

**Methods:**

To assess the DNA methylation status of the *Rb *promoter, genomic DNA from stably transfected human erythroleukemic K562 cells expressing a *GFP *reporter transgene was transformed with sodium bisulfite, and then PCR-amplified with modified primers and sequenced. Single- and multi-copy integrants with the CTCF binding site mutated were isolated and characterized by Southern blotting. Silenced transgenes were reactivated using 5-aza-2'-deoxycytidine and Trichostatin-A, and their expression was monitored by fluorescent cytometry. *Rb *gene expression and protein abundance were assessed by RT-PCR and Western blotting in three different glioma cell lines, and DNA methylation of the promoter region was determined by sodium bisulfite sequencing, together with CTCF dissociation and methyl-CpG-binding protein incorporation by chromatin immunoprecipitation assays.

**Results:**

We found that the inability of CTCF to bind to the *Rb *promoter causes a dramatic loss of gene expression and a progressive gain of DNA methylation.

**Conclusions:**

This study indicates that CTCF plays an important role in maintaining the *Rb *promoter in an optimal chromatin configuration. The absence of CTCF induces a rapid epigenetic silencing through a progressive gain of DNA methylation. Consequently, CTCF can now be seen as one of the epigenetic components that allows the proper configuration of tumor suppressor gene promoters. Its aberrant dissociation can then predispose key genes in cancer cells to acquire DNA methylation and epigenetic silencing.

## Background

DNA methylation and histone post-translational modifications have been considered as the main processes involved in conferring plasticity to transcriptional programs, and for allowing the transmission of epigenetic traits, including those involved in certain diseases [1]. Aberrant DNA methylation remains a central component of tumor suppressor gene epigenetic silencing in cancer, but the causes of abnormal methylation remain unclear [2]. Potential clarification is emerging from genome-wide surveys that determine different features of promoters known to contain CpG-islands, which are classified according to their high, intermediate and low CpG content [3,4]. Different degrees of DNA methylation cause diverse effects depending on the sub-class of CpG-island present. More recently, these studies have been complemented by a novel concept: the "CpG-island shores" [5]. A CpG-island shore refers to the way in which a CpG-island incorporates DNA methylation. Interestingly, this propagates from opposite ends, moving forward to the central region, which is frequently composed of the highest CpG dinucleotide density [5]. This evidence suggests that DNA methylation does not initiate necessarily where the highest CpG density is located, but instead, the abnormal methylation starts from the "outside" towards the "inside" by unknown mechanisms over promoter regions. Additional consequences emerge from these observations: 1) It is difficult to define where DNA methylation begins at a specific CpG-island; and 2) It is probable, as anticipated, that a critical density of methylated CpGs are needed for the epigenetic silencing to occur.

Conversely, there is also evidence suggesting that promoter regions associated with tumor suppressor genes, corresponding to CpG-islands, are not necessarily silenced by DNA methylation [6]. Indeed, the genomic context and even larger genomic segments like "long range epigenetic silencing" (LRES) regions can negatively modify the chromatin structure of gene promoters within these regions that are linked to cancer [7]. In spite of all this evidence, the mechanisms of epigenetic silencing by DNA methylation are not completely understood.

We have previously analyzed the promoters of the human *retinoblastoma *(*Rb*) and *p53 *tumor suppressor genes, where these promoters correspond to high CpG and low CpG islands, respectively [8,9]. We have found that the *Rb *promoter is mainly silenced by DNA methylation [8]. In contrast, the *p53 *core promoter does not acquire DNA methylation, and instead, it shows the incorporation of the repressive histone mark H3K27me3 in glioblastoma cell lines [9]. In both cases, we demonstrated that the multifunctional nuclear factor CTCF seems to shield the promoters against epigenetic silencing. Interestingly, these results are not restricted to the *Rb *and *p53 *genes, since similar observations have been reported for the human *BRCA1 *and *p16^INK4a ^*genes [10-12].

With the aim of better understanding the mechanisms associated with *Rb *promoter epigenetic silencing, we analyzed its DNA methylation status in detail in different cell types [13-16]. We asked whether the absence of CTCF binding to the *Rb *promoter is able to contribute, firstly, to more rapid and extensive DNA methylation, and secondly, to faster and robust epigenetic silencing. Indeed, we found that the inability of CTCF to bind to its recognition motif at the human *Rb *promoter causes accelerated DNA methylation and epigenetic silencing in transgenes and in glioma cells. Our results demonstrate that CTCF is a key component of a sub-class of gene promoters, and that its deregulation may be one of the steps leading to cancer development.

## Methods

### Cell cultures

HeLa cells were cultured in DMEM medium; K562 cells were cultured in ISCOVE medium; human glioblastoma-astrocytoma U87MG and U373MG cells, neuroblastoma SHSY-5Y cells, and human glioblastoma T98G cells were cultured in RPMI-1640 medium; all media contained 10% (v/v) fetal bovine serum (FBS) and 1% penicillin/streptomycin. Culture media were purchased from Invitrogen. Cells were maintained at 37°C in a humidified 5% CO_2_-containing atmosphere.

### Plasmids

The *Rb *promoter genomic DNA (positions 1634-2020, GenBank accession number L11910) was subcloned into the XhoI/HindIII sites of pGL3-Basic plasmid (Promega) to generate the pGLRb plasmid. The pERb and pERbmutE plasmids containing the GFP reporter gene under the control of the *Rb *wild-type and mutated promoters, respectively, were previously generated and described by De La Rosa-Velazquez *et al*., 2007 [8]. The integrity of all plasmid constructs was verified by DNA sequencing.

### Stable transfection of K562 cells and reactivation assay

Linearized pERb and pERbmutE were used to generate stable cell lines. After selection, neomycin-resistant clones were isolated and analyzed by fluorescence-activated cell sorting (FACS). Clones were subsequently cultured in the absence of neomycin in medium for up to 23 weeks. The integrity of the transgene was tested by Southern blotting. For reactivation experiments, stable cell lines were treated for 3 days with 5-aza-2'-deoxycytidine (30 μmol/ml), Trichostatin-A (15 ng/ml), or both. After treatment, the cells were harvested in FACS flow (BD Science) and analyzed by FACS. The percentage of reactivated cells was plotted in the corresponding graph.

### Sodium bisulfite treatment and methylation analysis

Bisulfite analysis was performed as described previously [9]. The PCR primers for stable cell lines were designed specifically against the plasmid sequences to avoid amplification of the endogenous promoter. Nested PCR was done with the EGFPbis1-EGFPbis2 primer pair, and the second round of PCR amplification was done with the EGFPbis3-EGFPbis4 primer pair. The product from the second PCR was gel-purified, and a third PCR was done with A3res-A4res primers specific for the *Rb *promoter (see below). PCR products were cloned in the pGEM-11zf vector (Promega) and sequenced using the T7 primer. Primers used were EGFPbis1: 5'-TTTGGTTTTTTGTTGGTT TTTTGT-3' and EGFPbis2: 5'-AAATAAACCAAAACACCAACAAC-3'; EGFPbis3: 5'-CG GGATCCTTTTTTTTGTGTTATTTTTTG-3' and EGFPbis4: 5'-CGGGATCCAAATCAACT TACCCTAAATAAC-3'; A3res: 5'-CGGGATCCTTAGGTTTTTTAGTTTAATTTTTTATGA T-3'; A4res: 5'-CGGGATCCAACTATAAAAAAACCCCAAAAAAAAC-3'; GFPRb-Fw: 5'-GGGATTTAGATTTTTTGTATAGTT-3' and GFPRb-Rev: 5'-CAAATAAACTTCAAAA TCAACTTAC-3'.

### Reagents and antibodies

The following antibodies were used in this study: acH3, acH4 antibodies, CTCF (N-17), MBD2 (N-18), MeCP2 (07-013) and Kaiso (clone 6F) from Millipore (Millipore); H3K27me3 antibody was kindly provided by Dr. Thomas Jenuwein (Max-Planck Institute of Immunobiology and Epigenetics, Freiburg, Germany). The rabbit anti-Rb (C-15) antibody (sc 12370) was purchased from Santa Cruz Biotechnology and horseradish peroxidase (HRP) linked to anti-rabbit immunoglobulin (81-6120) from Zymed. Mouse anti-human actin antibody was kindly provided by Dr. Alejandro Zentella Dehesa (Instituto de Investigaciones Biomédicas, UNAM, México).

### Semiquantitative RT-PCR and Western blot analysis

For semiquantitative RT-PCR, total RNA was isolated using Trizol reagent from Invitrogen Corporation (GIBCO), and 5 μg RNA from each preparation was used as an RT template in a reverse transcription system (Promega). PCR was performed using the following specific primer pairs: Ex27RBF2: 5'-GGTATGTAACAGCGACCGTGTG-3' and Ex27RBRev: 5'-CTCTTCCTTG TTTGAGGTATCC-3'; β-actin: 5'-CGTACCACTGGCATCGTG-3' and 5'-GGTAGTCAGTC AGGTCCC-3'. Total protein concentration was determined using the commercial Bradford reagent assay (Bio-Rad). 10 μg of total protein was used for the detection of Rb. Samples were first boiled in sample buffer (125 mM Tris-HCl pH 6.8, 1% v/w SDS, 10% v/v glycerol, 0.1% bromophenol blue, 2% v/v 2β-mercaptoethanol) for 5 min and resolved by 10% SDS-PAGE. The proteins were transferred to PVDF membranes (Bio-Rad) using a Trans-Blot Cell system (Bio-Rad) in transfer buffer (25 mM Tris, 190 mM glycine, 10% methanol) at 40 V overnight. The following day, the membrane was probed for 1 hr with rabbit anti-Rb (C-15) antibody diluted 1:2000 in TBS buffer (150 mM NaCl, 20 mM Tris, 0.1% Tween, 1% BSA, 5% non fat milk, pH 7.5). After extensive washing, the membrane was incubated for 1 hr with HRP anti-rabbit immunoglobulin. The signals were detected by enhanced chemiluminescence using the supersignal system (Pierce) and quantified by densitometry. As a control, actin was simultaneously detected using a mouse anti-human actin antibody diluted 1:750, and then developed using horseradish peroxidase linked to anti-mouse immunoglobulin and the same chemiluminescence system.

### Chromatin immunoprecipitation assay

ChIP assays were performed as described previously [8]. Briefly, U87MG glioma cells (2 × l0^7 ^cells) were fixed with 1% formaldehyde and then neutralized by adding 0.125 M glycine. Cells were collected and lysed in Cell Lysis buffer (5 mM EDTA pH 8.0, 50 mM de TRIS-HCl pH 8.1, 1% SDS, protease inhibitor cocktail). The nuclei lysate was sonicated to obtain soluble chromatin with an average length of 500 bp. After 1:10 dilution with dilution buffer (20 mM Tris-Cl, pH 8.1, 2 mM EDTA, 150 mM NaCl, 1% Triton X-100), chromatin solutions were precleared and then incubated with or without 4 μg anti-CTCF, anti-acH3, anti-acH4 and anti-H3K27me3 antibodies, then the mixture was incubated at 4°C overnight on a rotating platform. The same immunocomplexes were recovered with protein A-Sepharose beads. After extensive washing, the bound DNA fragments were eluted, and the resulting DNA was subjected to PCR reactions using the following primer pairs: RbPromFw/RbPromRev for the endogenous *Rb *promoter and RbPromFw/Hec02r for the stable transgene. As a negative control primers RTRb-F/RTRB-R were used to amplify exon 27 of the *Rb *gene. PCR products were separated by gel electrophoresis on a 2% agarose gel and visualized. Primer used: RbPromFw: 5'-CCAGACTCTTTGTATAGCC-3'; HEC02R: 5'-ACCATGGTGGCGACC-3'; RTRb-F: 5'-AAGTACCCATCTAGTACT-3'; RTRb-R: 5'-AAGTTACAGCATCTCTAAA-3'; Igf2-CTCF-Fwd: 5'-CAGGCTCCCCCAAAATCTA-3'; Igf2-CTCF-Rvs: 5'-GGGAACATAGAGAAAGAGG-3'; Retinoblastoma Exon 27: RTRb-F 5'-AAGTACCCATCTAGTACT-3' and RTRb-R: 5'-AAGTTACAGCATCTCTAAA-3' [8].

## Results

### *Retinoblastoma *promoter DNA methylation increases over time in cell culture

**Figure 1:**
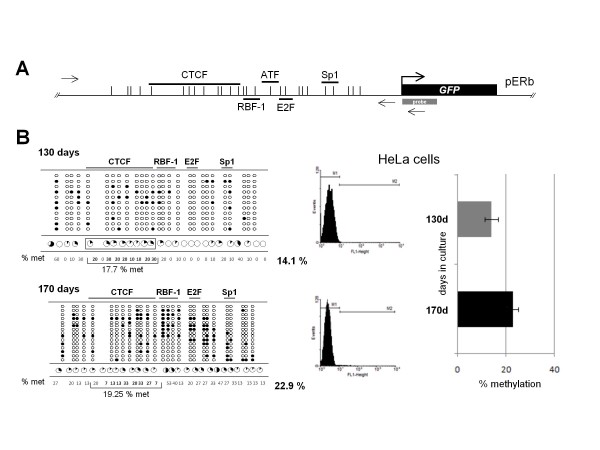
**Progressive gain of DNA methylation over the human retinoblastoma promoter**. A, Scheme of the transgene reporter carrying the core *Rb *promoter. The reporter gene (black rectangle) corresponds to the *GFP *gene, and locations of the relevant transcription factor binding sites including that of CTCF are shown. The vertical lanes correspond to the CpG dinucleotides. Locations of primers for PCR amplification of the bisulfite modified genome are shown as horizontal arrows. The probe used for Southern blots to validate the integrity of the transgenes and to determine copy number is also shown. B, DNA methylation status of a HeLa cell line in which the transgene shown in A is integrated as a single-copy. The individual and global percentages of methylation are shown at 130 and 170 days of continuous cell culture (left panel). As shown in a previous study [8], at 100 days in culture there is no expression of the transgene, but here we clearly observe a progressive increase in DNA methylation over the *Rb *promoter. At the center, the flow cytometry profile of the clone is shown at the same days, where 10,000 cells were analyzed. The intensity of fluorescence was plotted; M1, corresponds to the non-fluorescent cell population, and M2, the fluorescent detectable part of the graph. The plot summarizing the percentage of DNA methylation is shown (right panel).

With the aim of further understanding the DNA methylation dynamics over the *Rb *promoter, we performed sodium bisulfite genomic DNA transformation and sequencing of the core promoter at 130 and 170 days of continuous cell culture (Figure 1A). The DNA methylation status was analyzed over a transgene that was stably integrated in HeLa cells [8]. Using the same cell line we had previously demonstrated a modest gain of DNA methylation (at 100 days in culture) with severe consequences on gene expression [8]. Here we found that after additional cell divisions there was a gradual but not dramatic increase in DNA methylation (Figure 1B). After 130 days, 14% DNA methylation was reached, and after 170 days, 22%. At this last time point we found an increase in the methylated CpG dinucleotides located preferentially over the CTCF, RBF-1 and E2F binding motifs (Figure 1B). These results suggest that after prolonged cell culture and many cell divisions there is a gain of DNA methylation, which reinforces epigenetic silencing.

### Faster epigenetic silencing of the *Rb *promoter with a mutated CTCF binding site

**Figure 2:**
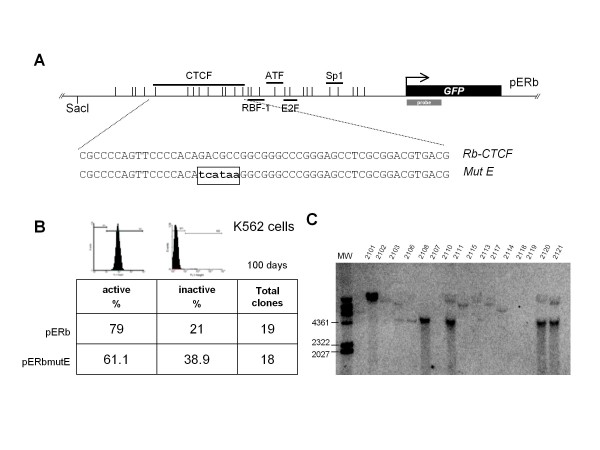
**Generation of stable lines with a mutated CTCF binding site**. A, The transgene and the location of the mutation E (mutE) are shown. B, Stable lines were generated in K562 human erythroleukemic cells and cultured for more than 100 days, with 19 independent clones carrying the intact promoter (pERb) and 18 independent clones with the core *Rb *promoter with the mutated CTCF binding site (pERbmutE). The point mutations were defined previously [8]. A representative FACS profile is shown for the active and inactive transgenes, respectively. These are results from two independent sets of transfections. C, A representative Southern blot is shown. For hybridization we used a 741 bp DNA probe corresponding to a portion of the *GFP *transgene. Genomic DNA from each cell line was digested with SacI restriction enzyme and the minimal hybridization signal corresponds to a band of 4,561 bp. This reference band allows us to determine the integrity of the transgene and the integration of a single- or a multi-copy transgene. MW, corresponds to the molecular marker λ-HindIII.

To confirm the contribution of CTCF to the protection of tumor suppressor gene promoters against epigenetic silencing, we decided to generate a large set of independent stably integrated constructs carrying the *Rb *promoter fused to the *GFP *reporter gene, where the promoter had a mutated CTCF site. The mutation (pERmutE) was previously defined and confirmed by gel-shift assays using probes carrying different mutations [8]. Nineteen independent lines with the intact promoter and 18 independent lines with the mutant version of the promoter were generated in the erythroleukemic K562 cell line (Figure 2A and 2B). Transgene copy-number and integrity was confirmed by Southern blotting (Figure 2C). For the transgene in which the CTCF binding site was mutated, we found an approximately 2-fold increase in the number of stable cell lines with extinguished transgene expression after 100 days of continuous cell culture (Figure 2). This transgene silencing was also clearly seen in FACS profiles at different time points (see Additional file 1, Figure S1). We conclude that CTCF binding to the *Rb *promoter is critical for its epigenetic integrity and transcriptional activity. This is in agreement with our previous CTCF knockdown experiments, where CTCF depletion caused a loss of *Rb *gene expression [9].

### Mutant CTCF-Rb promoter transgenes are differentially reactivated

**Figure 3:**
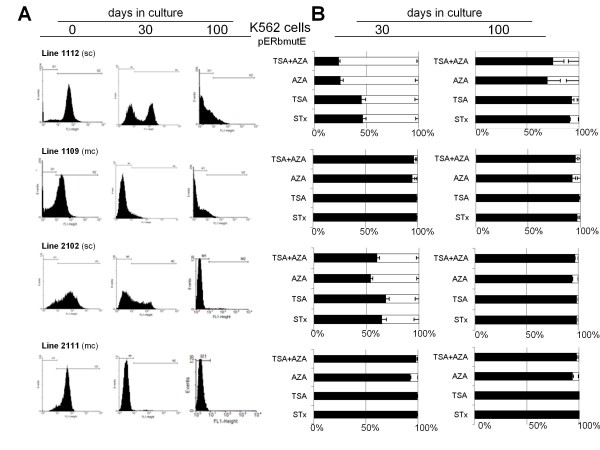
**The CTCF binding site mutant at the *Rb *promoter is differentially reactivated**. A, Flow cytometry profiles of four K562 stable cell lines expressing the transgene described in 2A after 0, 30 and 100 days of continuous cell culture. These lines were isolated and selected from two independent experiments; sc, single-copy integrant, and mc, multi-copy integrants. B, Reactivation experiments of the corresponding cell lines performed at 30 days and 100 days of continuous cell culture. Reactivation assays were performed using 5-aza-2'-deoxycytidine (AZA), trichostatin-A (TSA) and mock cells (STx). The plot represents the average of fluorescent cells, where the black bar corresponds to the inactive cell population and the white bar to the fluorescent cell population. The results correspond to the average of three independent reactivation experiments for each cell line, and the standard error is shown.

Taking advantage of the K562 stable cell lines carrying a mutated *Rb *promoter (pERbmutE: lines 1109, 1112, 2102 and 2111), we performed reactivation experiments using the histone deacetylase inhibitor Trichostatin-A (TSA), the DNA methylation inhibitor 5-aza-2'-deoxycytidine (5-azadC), and their combination (Figure 3A). Reactivation conditions were previously established (Additional file 2, Figure S2) and transgene activity was followed at 30 and 100 days of continuous cell culture. Interestingly, we found that a significant reactivation was observed at early time points of cell culture (30 days) using each of the inhibitors or their combination. A more pronounced reactivation was observed when the 5-azadC inhibitor was tested compared to TSA treatment (Figure 3B). Of note, single-copy integrants were more efficiently reactivated in comparison to multi-copy integrants (Figure 3B). This observation may be related to evidence demonstrating that multi-copy integrants can be recognized as repetitive sequences that induce the formation of heterochromatin [17-19]. Unexpectedly, at 100 days in cell culture the reactivation became modest. The DNA methylation status of the reactivated cell line 1112 and 2111, and a new and independent cell line 2102, were assessed (Additional file 3, Figure S3). Thus, in these series of reactivation experiments, using the 5-azadC inhibitor, DNA methylation was partially lost indirectly demonstrating that DNA methylation is one of the causes of silencing.

These results could appear contradictory to our previous observations where we observed 60% reactivation using 5-azadC in a HeLa cell line [8], but this seems not to be the case since that cell line, which carried an intact CTCF sequence, was previously sorted from a clone that was partially silenced after 100 days in continuous cell culture [8]. Additionally in the same previous work, we found similar reactivation levels at early time points, as observed here in the context of the mutant CTCF sites, which are more rapidly and robustly silenced. These results exemplify two different processes leading to the establishment of an epigenetic silencing conformation over the *Rb *core promoter. We believe that at early time points (30 days), DNA methylation is progressively and actively incorporated without reaching critical densities or resulting in the methylation of specifically located CpG dinucleotides. This is likely because this sort of intermediate state of DNA methylation can be efficiently reverted. In contrast, at later times of cell culture (100 days or more), histone deacetylation appears to be irreversible, but DNA methylation is partially erased suggesting that other repressive histone marks and nuclear factor complexes have been established. One attractive possibility is the gain of the histone H3K27me3 mark, a repressive mark incorporated by members of the Polycomb group repressor proteins.

### DNA hypermethylation of the *Rb *promoter in a transgene with a mutated CTCF binding site

**Figure 4:**
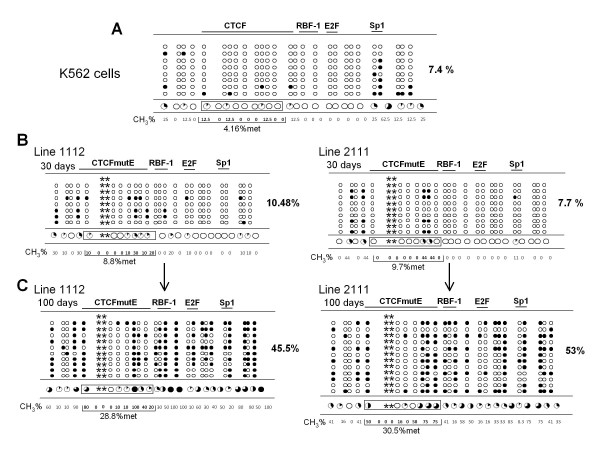
**Cell lines with a mutated CTCF binding site are DNA hypermethylated**. A, DNA methylation analysis of the wild-type, transcriptionally active *Rb *core promoter at 100 days of cell culture. B and C, Sodium bisulfite analysis of stable lines 1112 and 2111 after 30 days and 100 days of continuous cell culture were assessed. The asterisks represent the mutated CpGs associated with the CTCF mutation E (CTCFmutE), as defined previously [8].

Next, we assessed the DNA methylation status of two of the stable cell lines generated with the mutated CTCF binding site. These cell lines were previously tested in the reactivation experiments (Figure 4; cell lines 1112 and 2111). For comparison we evaluated the DNA methylation over a transgene in a K562 stable line in which the *Rb *promoter was intact (Figure 4A). In agreement with previous observations, we found that there was no significant DNA methylation at day 30 of continuous cell culture even though transgene expression started to decay (see Figure 4B). Instead, after 100 days in culture the 1112 and 2111 cell lines became DNA hypermethylated (Figure 4C). This result is consistent with the inability of CTCF to bind to the transgene *Rb *promoter. This was further confirmed by bisulfite sequencing of the K562 endogenous *Rb *promoter, where we found less than a 2-fold gain of DNA methylation (Additional file 4, Figure S4). Taken together, these results demonstrate that CTCF is a critical factor that contributes to the epigenetic protection of the *Rb *core promoter, which in the absence of CTCF binding, is prone to DNA hypermethylation.

### CTCF absence correlates with *Rb *promoter epigenetic silencing in glioma cells

**Figure 5:**
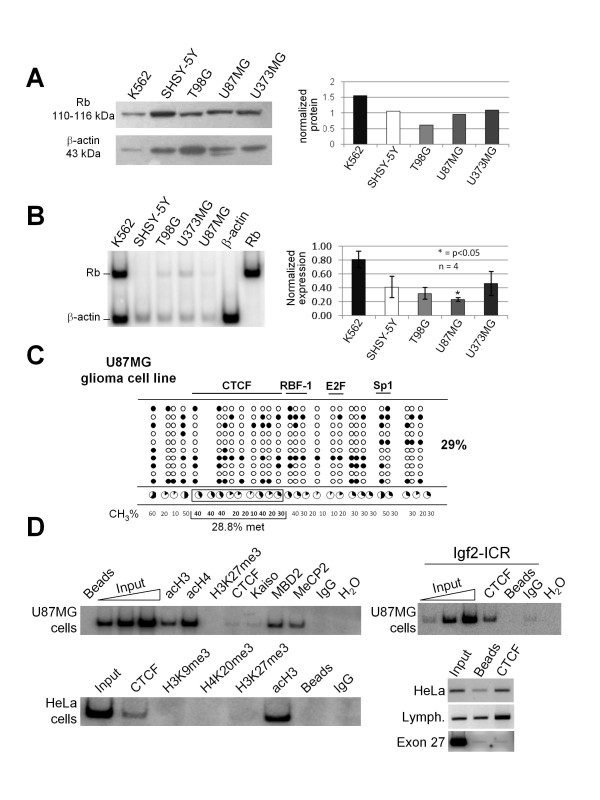
**Epigenetic silencing of the *Rb *promoter in glioma cells**. A, Western blot of the Rb protein in four different glioma cell lines and erythroleukemic K562 cells. The blot was re-incubated with a human β-actin antibody for normalization and the resulting data were plotted (right panel). B, RT-PCR assay using radioactive duplex-PCR for semi-quantitative transcription level determination was performed. The results of four independent experiments are plotted and standard errors are shown. C, DNA methylation status of the U87MG glioma cells was determined by sodium bisulfite sequencing. D, Chromatin immunoprecipitation assay from U87MG glioma cells, HeLa cells and human quiescent lymphocytes. As a positive control, the *in vivo *CTCF binding at the Imprinting Control Region of the *Igf2/H19 *(Igf2-ICR) imprinted locus is shown in U87MG glioma cells. Additional controls are shown using HeLa cells. As a negative control we used primers covering the *Rb *Exon 27 (546 bp product, with genomic position +175819 to +176365) located ~200 kb downstream of the *Rb *promoter. The linear range of amplification is shown as Input for the U87MG series of ChIPs, and IgG was used as a non-specific antibody.

To further assess the contribution of CTCF to the protection of the *Rb *promoter against DNA methylation, we analyzed a series of glioma cell lines (Figure 5). Based on our previous observations in which CTCF was not bound to the *p53 *promoter *in vivo*, we asked if in glioma cells the lack of *Rb *gene expression could correlate with CTCF absence at the *Rb *promoter [9]. We first evaluated the relative concentration of Rb by Western blotting in four different glioma cell lines (SHSY-5Y, T98G, U87MG and U373MG) compared to human K562 cells (Figure 5A). In this experiment we noticed that T98G and U87MG cells had the lowest amount of Rb protein compared to the other transformed cell lines. Next, we determined *Rb *mRNA levels by semi-quantitative duplex-PCR (Figure 5B). *Rb *transcription was significantly diminished in the U87MG glioma cell line. Based on these results we performed sodium bisulfite sequencing and sequencing of the endogenous *Rb *core promoter in the U87MG cell line (Figure 5C). In agreement with the *Rb *gene expression levels, we found that the *Rb *promoter reached 29% DNA methylation in U87MG cells. Subsequently, we performed a chromatin immunoprecipitation assay employing primers to amplify the *Rb *core promoter and a set of antibodies against different histone marks, CTCF and methyl-CpG-binding proteins. The results revealed no decrease in histone acetylation, gain of the H3K27me3 histone repressive mark, incorporation of the methyl-CpG-binding proteins MBD2 and MeCP2, and importantly, we confirmed the absence of CTCF bound to the *Rb *promoter in U87MG cells (Figure 5D). As a positive control for the *in vivo *binding of CTCF, we performed a ChIP assay in U87MG glioma cells using primers from the Imprinting Control Region of the human *Igf2/H19 *imprinted locus, which is known to have several CTCF binding sites [20,21]. In summary, these results support our *in vitro *data and demonstrate that in a tumoral cell line, the lack of CTCF binding to the *Rb *promoter is, in part, responsible for its epigenetic silencing.

## Discussion

The methylation of DNA is considered to be one of the most relevant processes in epigenetics and is tightly associated with cancer. Several aspects have evolved concerning the CpG-island features and the chromatin remodeling activities linked to tumor suppressor gene epigenetic silencing, but up until now, selective predisposition to abnormal DNA methylation has been poorly understood. Based on the hypothesis that genomic boundaries may contribute to the shielding of CpG-island promoters against silencing, we investigated the contribution of CTCF and DNA methylation to the epigenetic regulation of the human *retinoblastoma *gene promoter. Indeed, we demonstrated that the dissociation of CTCF from its recognition sequence causes an increase in DNA methylation and the rapid gain of a repressive chromatin configuration over the *Rb *promoter.

The concept of genomic boundaries and the propagation of repressive chromatin structures from nucleation sites has been suggested previously [22]. Data from our research group supports this view on the basis of the multifunctional activity of the 11-zinc-finger nuclear factor CTCF [11,23]. The involvement of CTCF in cancer and in particular, in the epigenetic regulation of tumor suppressor genes, is further supported by recent data from other laboratories studying the *BRCA1 *and *p16^INK4a ^*tumor suppressor genes, or even the apoptotic gene *PUMA *[8-10,12,24]. In addition, CTCF has been proposed to participate in genome organization through lamin and cohesin interactions, and in delineating transitions between open and repressive chromatin regions at the genome-wide scale [25,26]. Thus, several lines of evidence, both at the local and genome-wide scales, support the contribution of CTCF to the optimal regulation of a significant number of genes.

Coincident with other studies, the mechanisms associated with gene silencing and reactivation assays remain controversial. An interesting result shown here is the capacity to partially reactivate silenced stable cell lines at early culture times, and the incapacity to achieve reactivation at later time points (Figure 3). These results are similar to those observed by Baylin and collaborators, where they concluded that DNA methylation inhibition does not reverse a repressive chromatin conformation and a silenced state in cancer cells [27,28]. In colorectal cancer cells the histone H3K27me3 repressive mark seems to be the preferential source of epigenetic silencing of the *hMLH1 *gene promoter [27]. Another possibility that can be independent of DNA methylation is the formation of higher order chromatin structures that can participate in gene repression [28]. The complexity of these processes is exemplified by the depletion of the Polycomb protein EZH2, which is responsible for the trimethylation of histone H3 lysine 27 and the persistence of DNA methylation. The variety of epigenetic silencing mechanisms appears to depend on the affected genes and the type of tumoral cell analyzed [29,30].

It is worth mentioning that we observe variations in the capacity to inhibit DNA methylation when we compare different stable lines after 30 days of continuous cell culture. For example, transgene expression of the 2111 cell line was rapidly silenced but no significant gain of DNA methylation was detected at 30 days of culture (Figure 3 and 4). In contrast, for the 1112 cell line a reactivation of the transgene was observed corresponding to around 10% de-methylation after 30 days in culture (Figure 3 and Additional file 3, Figure S3). These data suggest that DNA methylation may not always be the initial silencing event. Several interpretations can be considered, among them, variations from line to line due to chromosomal position effects caused by different transgene integration sites. Extrapolating these findings to the endogenous context, one possibility is that more distal genomic sequences may possibly gain DNA methylation that causes, either directly or indirectly, *Rb *gene silencing in the absence of CTCF binding [31]. We also cannot discard the possibility that DNA methylation is affecting the expression of a co-regulator of CTCF, such as the poly-ADP-ribosyl polymerase 1, among others [32].

However, DNA methylation does not seem to be the only source of silencing. We postulate that the features of the CpG-island associated with each promoter can dictate its mechanism of silencing and that CTCF protects those promoters in the absence of DNA methylation, like in the case of the human *p53 *gene promoter [9]. Another aspect that remains unexplored is the reason for aberrant CTCF function. One scenario could be related to the post-translational modifications that CTCF is subject to, like phosphorylation and poly(ADP-ribosyl)ation [32]. Interestingly, it has been recently demonstrated that CTCF and PARP-1-dependent poly(ADP-ribosyl)ation can induce DNA hypomethylation by inhibiting the DNA methyltransferase Dnmt1 [33]. Thus, in cancer a deregulation of CTCF-PARP-1 poly(ADP-ribosyl)ation levels may cause the activation of Dnmt1 and the local hypermethylation of the *Rb *promoter.

Our results show that over long periods of time, other repressive mechanisms in addition to DNA methylation can participate in *Rb *promoter epigenetic silencing. We postulate that non-coding RNAs can perform such role, acting in *cis *or *trans*, like the long non-coding RNAs HOTAIR and lincRNA-p21 [34,35].

## Conclusion

CTCF plays an important role in maintaining regulatory regions of certain genes in optimal chromatin configurations. On the basis of the data accumulated by our group and other laboratories, it is now critical to begin addressing mechanistic questions concerning the aberrant performance of CTCF in cancer cells. Moreover, it is time to go forward and consider CTCF as a potential tumor suppressor gene or molecular marker for different types of tumors.

## Abbreviations

The abbreviations used are: *CTCT*: CCCTC-binding factor; *ChIP*: Chromatin immunoprecipitation; *Rb*: retinoblastoma gene; *GFP*: Green Fluorescent Protein.

## Competing interests

The authors declare that they have no competing interests.

## Authors' contributions

MDS and FRT designed the experiments. MDS, MFM and FRT wrote and revised the manuscript. MDS performed the majority of the experiments. CVQ and EAO performed CIP assays. EGB and CVQ performed cell culture and expression experiments. All authors read and approved the final manuscript.

## Pre-publication history

The pre-publication history for this paper can be accessed here:

http://www.biomedcentral.com/1471-2407/11/232/prepub

## Supplementary Material

Additional file 1 **Figure S1. Flow cytometry profiles of stably transfected K562 cells expressing the *GFP *reporter gene under the control of the *Rb *promoter with a wild-type and mutated CTCF binding site**. Individual lines carrying the pERb transgene (left panel) that includes the intact *Rb *promoter (as shown in Figure 1A and 2A), and the same transgene with the CTCF binding site mutated, pERbmutE, are shown (right panel). Each individual cell line was isolated in soft-agar in the presence of drug-selection and the integrity of the transgene for each cell line was confirmed by Southern blotting, as described in the legend for Figure 2C. Single-copy and multi-copy integrants were determined in this way. Note that for the intact *Rb *promoter the great majority of established cell lines are robustly active even after 100 days of continuous cell culture. Few exceptions are found, like line 012 in which the transgene is probably subject to a strong repressive effect due to its genomic integration site, but in general we consider the *Rb *promoter to be a "strong" promoter. When the CTCF binding sequence is mutated (pERbmutE), a rapid expression extinction of the transgene is observed with, in addition, more variable levels of expression, suggesting that the transgene is more prone to chromosomal position effects under these conditions. Click here for file

Additional file 2 **Figure S2. Standardization of the DNA methylation and histone deacetylation inhibitor concentrations**. Representative FACS profiles are shown with the corresponding inhibitor concentrations. Graphs summarizing the percentage of *GFP *expression reactivation are shown. Click here for file

Additional file 3 **Figure S3. DNA methylation status of the human *Rb *promoter after 5-azadC inhibitor treatment**. To analyze the degree of de-methylation, we analyzed the stably transformed K562 cell line 2102 (mc; single-copy) and line 1112 (sc; single-copy), which were maintained in continuous cell culture for 100 days. We isolated genomic DNA from each cell line and performed sodium bisulfite sequencing. Click here for file

Additional file 4 **Figure S4. DNA methylation analysis of the endogenous human *Rb *promoter**. A, For comparison purposes we used a wild-type *Rb *promoter-GFP transgene in K562 cells that is transcriptionally active after 100 days of cell culture. B, Endogenous DNA methylation status of the *Rb *promoter in the K562 cell lines 1112 and 2111 at 30 days of cell culture. C, This panel is similar to B and corresponds to both cell lines at 100 days of cell culture. Individual (for each CpG) and global DNA methylation percentages are indicated. Click here for file
