# Supplementary figures and images for: Gain of DNA methylation is enhanced in the absence of CTCF at the human retinoblastoma gene promoter
Source: BMC Cancer. 2011 Jun 10;11:232. doi: 10.1186/1471-2407-11-232 (PMC3145615; doi:10.1186/1471-2407-11-232)

## Slide 1
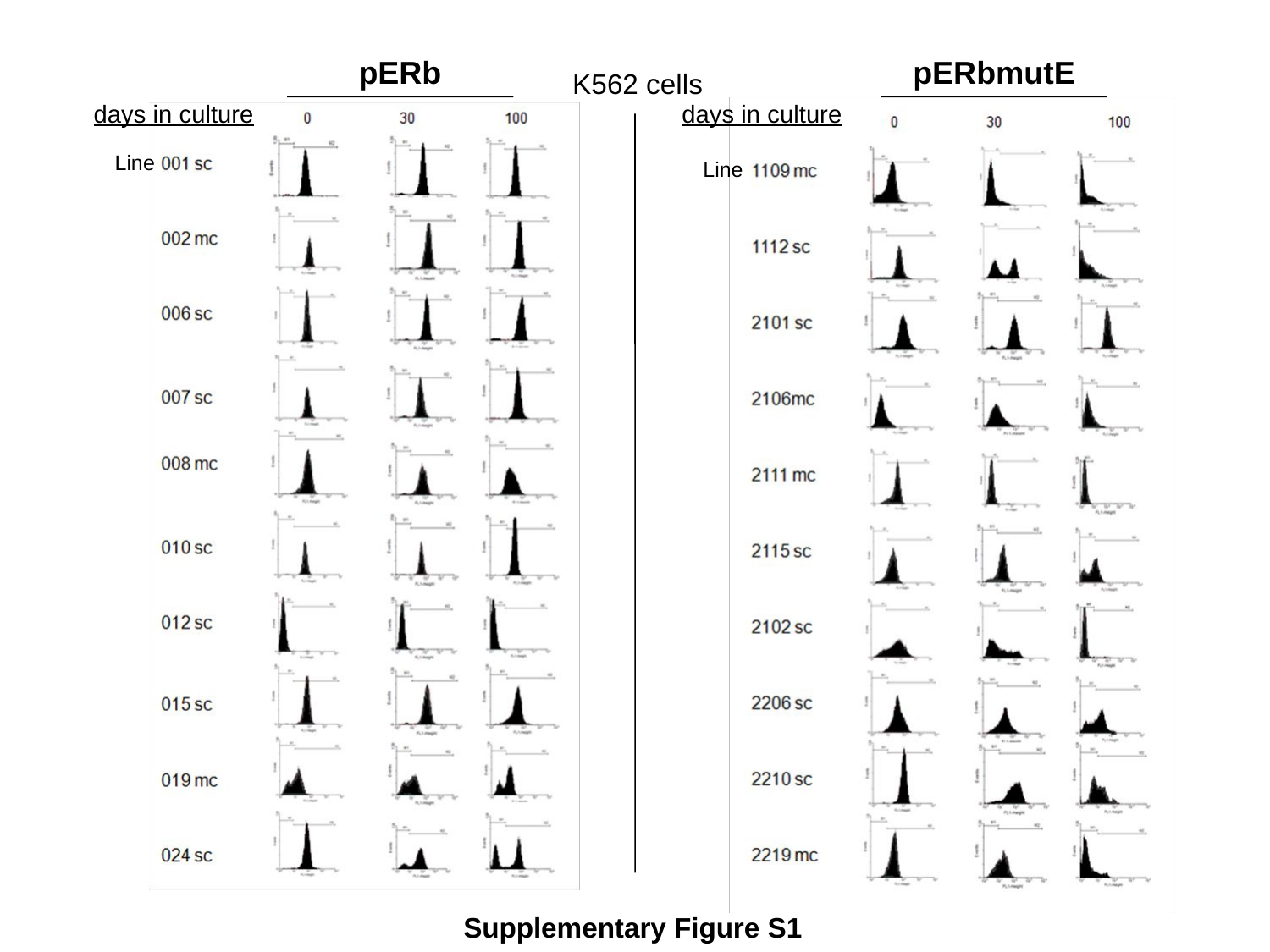

pERb
pERbmutE
K562 cells
days in culture
days in culture
Line
Line
Supplementary Figure S1

Supplement: Additional file 1 — Figure S1. Flow cytometry profiles of stably transfected K562 cells expressing the GFP reporter gene under the control of the Rb promoter with a wild-type and mutated CTCF binding site. Individual lines carrying the pERb transgene (left panel) that includes the intact Rb promoter (as shown in Figure 1A and 2A), and the same transgene with the CTCF binding site mutated, pERbmutE, are shown (right panel). Each individual cell line was isolated in soft-agar in the presence of drug-selection and the integrity of the transgene for each cell line was confirmed by Southern blotting, as described in the legend for Figure 2C. Single-copy and multi-copy integrants were determined in this way. Note that for the intact Rb promoter the great majority of established cell lines are robustly active even after 100 days of continuous cell culture. Few exceptions are found, like line 012 in which the transgene is probably subject to a strong repressive effect due to its genomic integration site, but in general we consider the Rb promoter to be a "strong" promoter. When the CTCF binding sequence is mutated (pERbmutE), a rapid expression extinction of the transgene is observed with, in addition, more variable levels of expression, suggesting that the transgene is more prone to chromosomal position effects under these conditions. [file 1471-2407-11-232-S1.PPT]
