# Supplementary material for: Gain of DNA methylation is enhanced in the absence of CTCF at the human retinoblastoma gene promoter
Source: BMC Cancer. 2011 Jun 10;11:232. doi: 10.1186/1471-2407-11-232 (PMC3145615; doi:10.1186/1471-2407-11-232)
Supplement: Additional file 2 — Figure S2. Standardization of the DNA methylation and histone deacetylation inhibitor concentrations. Representative FACS profiles are shown with the corresponding inhibitor concentrations. Graphs summarizing the percentage of GFP expression reactivation are shown. [file 1471-2407-11-232-S2.PPT]

## Slide 1
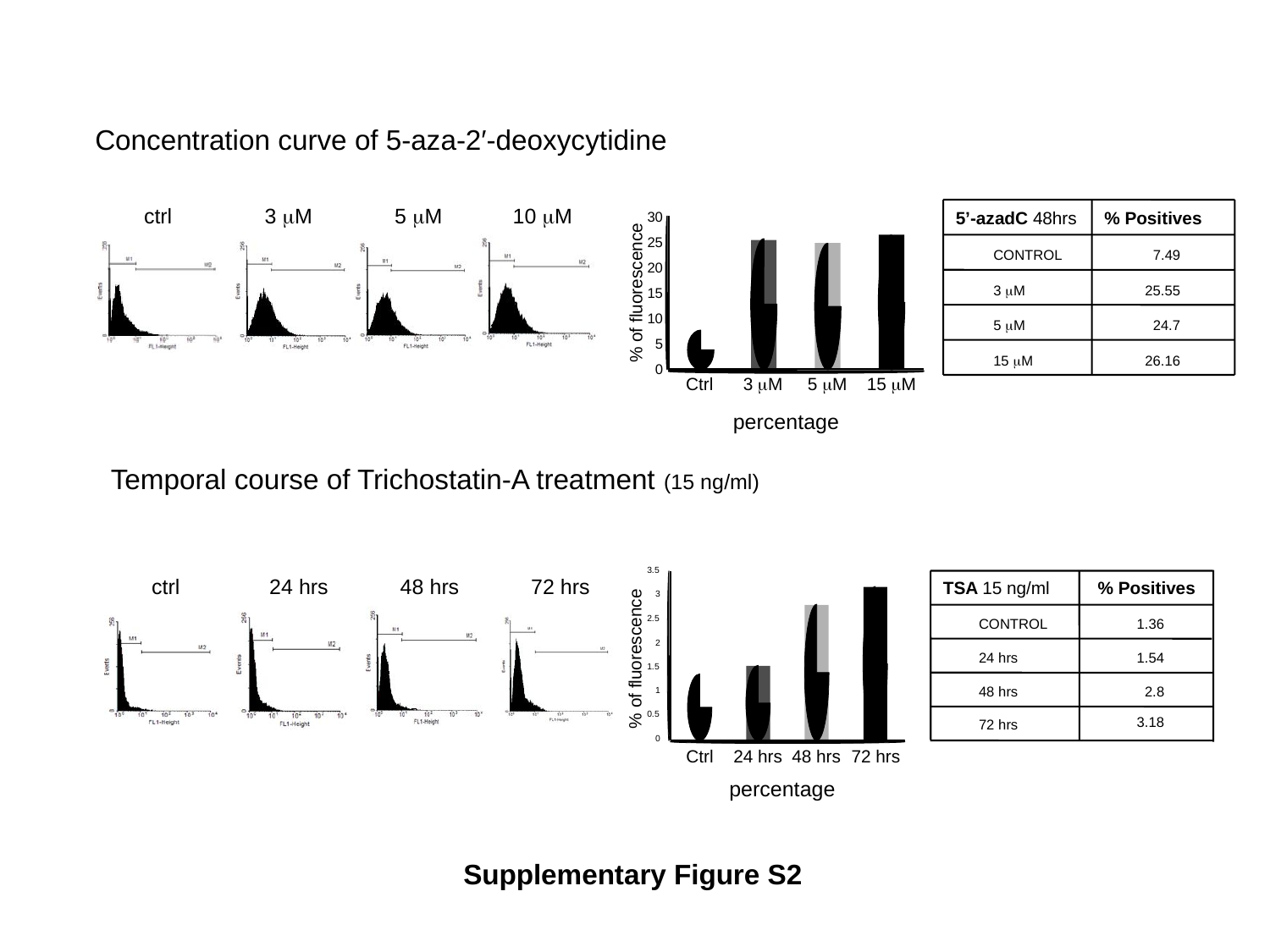

Concentration curve of 5-aza-2′-deoxycytidine
ctrl
3 μM
5 μM
10 μM
5’-azadC 48hrs
% Positives
30
25
CONTROL
7.49
20
3 μM
25.55
% of fluorescence
15
5 μM
24.7
10
5
15 μM
26.16
0
Ctrl
3 μM
5 μM
15 μM
 percentage
Temporal course of Trichostatin-A treatment (15 ng/ml)
3.5
ctrl
24 hrs
48 hrs
72 hrs
TSA 15 ng/ml
% Positives
3
CONTROL
1.36
2.5
2
24 hrs
1.54
% of fluorescence
1.5
48 hrs
2.8
1
72 hrs
3.18
0.5
0
Ctrl
24 hrs
48 hrs
72 hrs
 percentage
Supplementary Figure S2
