# Supplementary material for: Gain of DNA methylation is enhanced in the absence of CTCF at the human retinoblastoma gene promoter
Source: BMC Cancer. 2011 Jun 10;11:232. doi: 10.1186/1471-2407-11-232 (PMC3145615; doi:10.1186/1471-2407-11-232)
Supplement: Additional file 3 — Figure S3. DNA methylation status of the human Rb promoter after 5-azadC inhibitor treatment. To analyze the degree of de-methylation, we analyzed the stably transformed K562 cell line 2102 (mc; single-copy) and line 1112 (sc; single-copy), which were maintained in continuous cell culture for 100 days. We isolated genomic DNA from each cell line and performed sodium bisulfite sequencing. [file 1471-2407-11-232-S3.PPT]

## Slide 1
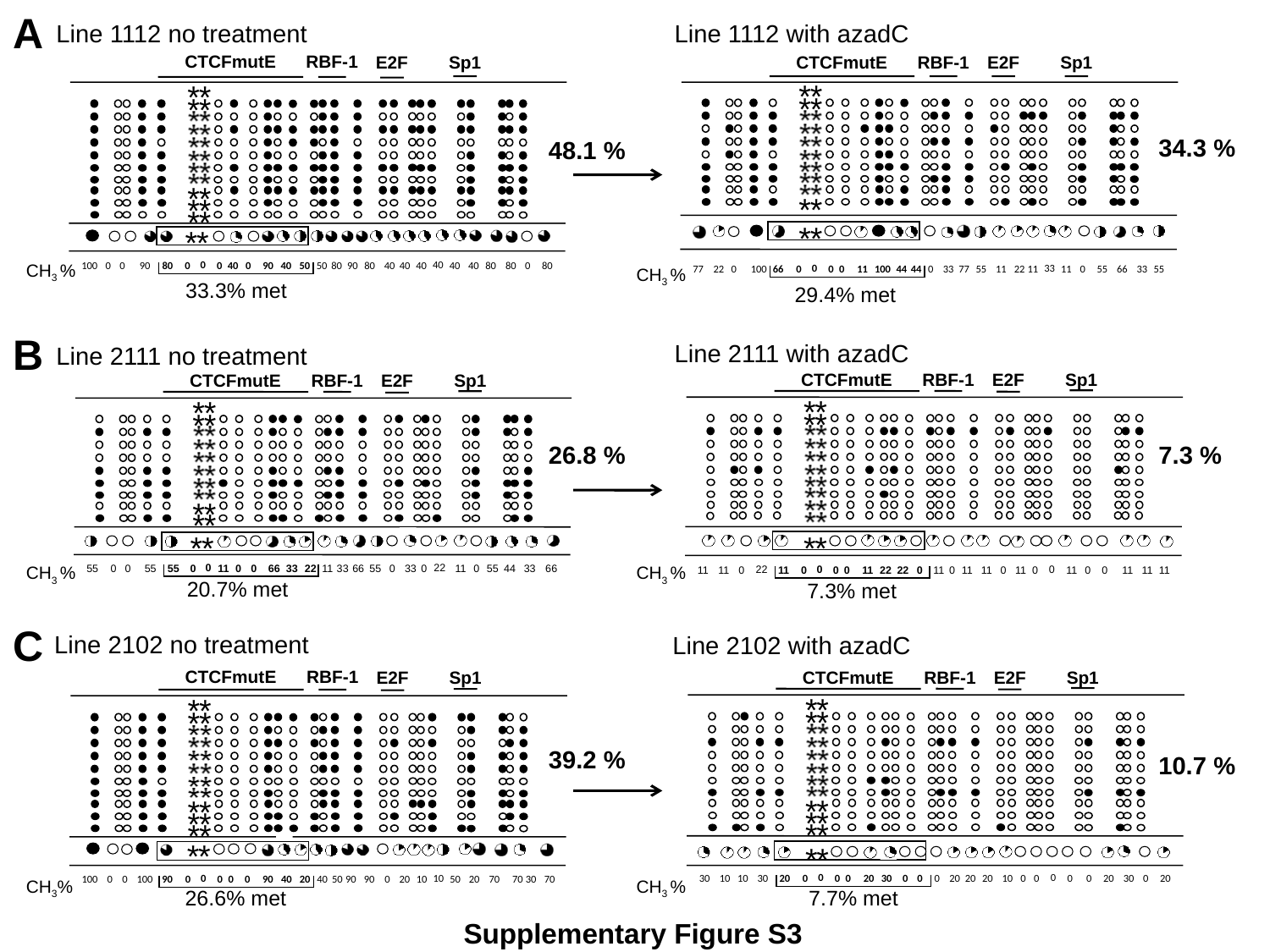

A
Line 1112 no treatment
Line 1112 with azadC
CTCFmutE
RBF-1
E2F
Sp1
CTCFmutE
RBF-1
E2F
Sp1
*
*
*
*
*
*
*
*
34.3 %
48.1 %
*
*
*
*
*
*
*
*
*
*
*
*
0
40
90
100
0
80
0
0
40
0
90
40
50
50
80
90
80
40
40
40
40
40
80
80
0
80
0
CH3 %
0
33
100
77
0
66
0
0
0
11
100
44
44
0
33
77
55
11
22
11
11
0
55
66
33
55
22
CH3 %
33.3% met
29.4% met
B
Line 2111 with azadC
Line 2111 no treatment
CTCFmutE
RBF-1
E2F
Sp1
CTCFmutE
RBF-1
E2F
Sp1
*
*
*
*
*
*
*
*
26.8 %
7.3 %
*
*
*
*
*
*
*
*
0
22
55
CH3 %
55
0
55
0
11
0
0
66
33
22
11
33
66
55
0
33
0
11
0
55
44
33
66
CH3 %
0
0
0
22
11
0
11
0
0
0
11
22
22
0
11
0
11
11
0
11
0
11
0
0
11
11
11
11
20.7% met
7.3% met
C
Line 2102 no treatment
Line 2102 with azadC
CTCFmutE
RBF-1
E2F
Sp1
CTCFmutE
RBF-1
E2F
Sp1
*
*
*
*
*
*
*
*
39.2 %
10.7 %
*
*
*
*
*
*
*
*
*
*
*
*
*
*
*
*
0
0
30
0
30
10
20
0
0
0
20
30
0
0
0
20
20
20
10
0
0
0
0
20
30
0
20
10
10
100
100
0
90
0
0
0
0
90
40
20
40
50
90
90
0
20
10
50
20
70
70
30
70
0
CH3%
CH3 %
26.6% met
7.7% met
Supplementary Figure S3
