# Supplementary material for: Gain of DNA methylation is enhanced in the absence of CTCF at the human retinoblastoma gene promoter
Source: BMC Cancer. 2011 Jun 10;11:232. doi: 10.1186/1471-2407-11-232 (PMC3145615; doi:10.1186/1471-2407-11-232)
Supplement: Additional file 4 — Figure S4. DNA methylation analysis of the endogenous human Rb promoter. A, For comparison purposes we used a wild-type Rb promoter-GFP transgene in K562 cells that is transcriptionally active after 100 days of cell culture. B, Endogenous DNA methylation status of the Rb promoter in the K562 cell lines 1112 and 2111 at 30 days of cell culture. C, This panel is similar to B and corresponds to both cell lines at 100 days of cell culture. Individual (for each CpG) and global DNA methylation percentages are indicated. [file 1471-2407-11-232-S4.PPT]

## Slide 1
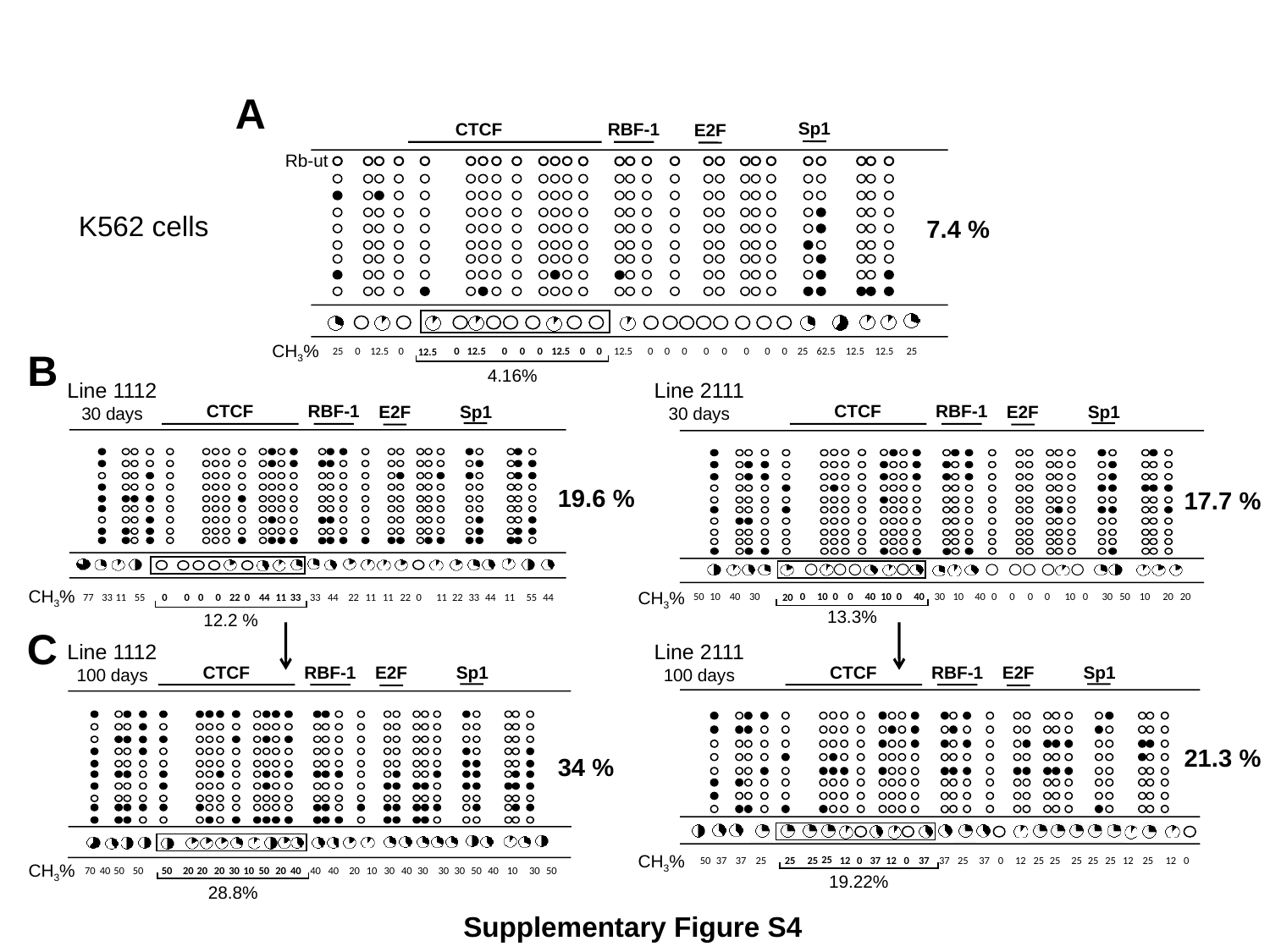

A
Sp1
CTCF
RBF-1
E2F
Rb-ut
K562 cells
7.4 %
CH3%
B
0
25
62.5
12.5
12.5
25
25
0
12.5
0
0
12.5
0
0
0
12.5
0
0
12.5
0
0
0
0
0
0
0
12.5
4.16%
Line 1112
30 days
Line 2111
30 days
CTCF
RBF-1
E2F
Sp1
CTCF
RBF-1
E2F
Sp1
19.6 %
17.7 %
CH3%
CH3%
50
10
40
30
0
10
0
0
40
10
0
40
30
10
40
0
0
0
0
10
0
30
50
10
20
20
77
33
11
55
0
0
0
0
22
0
44
11
33
33
44
22
11
11
22
0
11
22
33
44
11
55
44
20
13.3%
12.2 %
C
Line 1112
100 days
Line 2111
100 days
CTCF
RBF-1
E2F
Sp1
CTCF
RBF-1
E2F
Sp1
21.3 %
34 %
CH3%
25
50
37
37
25
25
25
12
0
37
12
0
37
37
25
37
0
12
25
25
25
25
25
12
25
12
0
CH3%
70
40
50
50
50
20
20
20
30
10
50
20
40
40
40
20
10
30
40
30
30
30
50
40
10
30
50
19.22%
28.8%
Supplementary Figure S4
